# Supplementary figures and images for: Pseudo-pac site sequences used by phage P22 in generalized transduction of Salmonella
Source: PLoS Pathog. 2024 Jun 24;20(6):e1012301. doi: 10.1371/journal.ppat.1012301 (PMC11226127; doi:10.1371/journal.ppat.1012301)

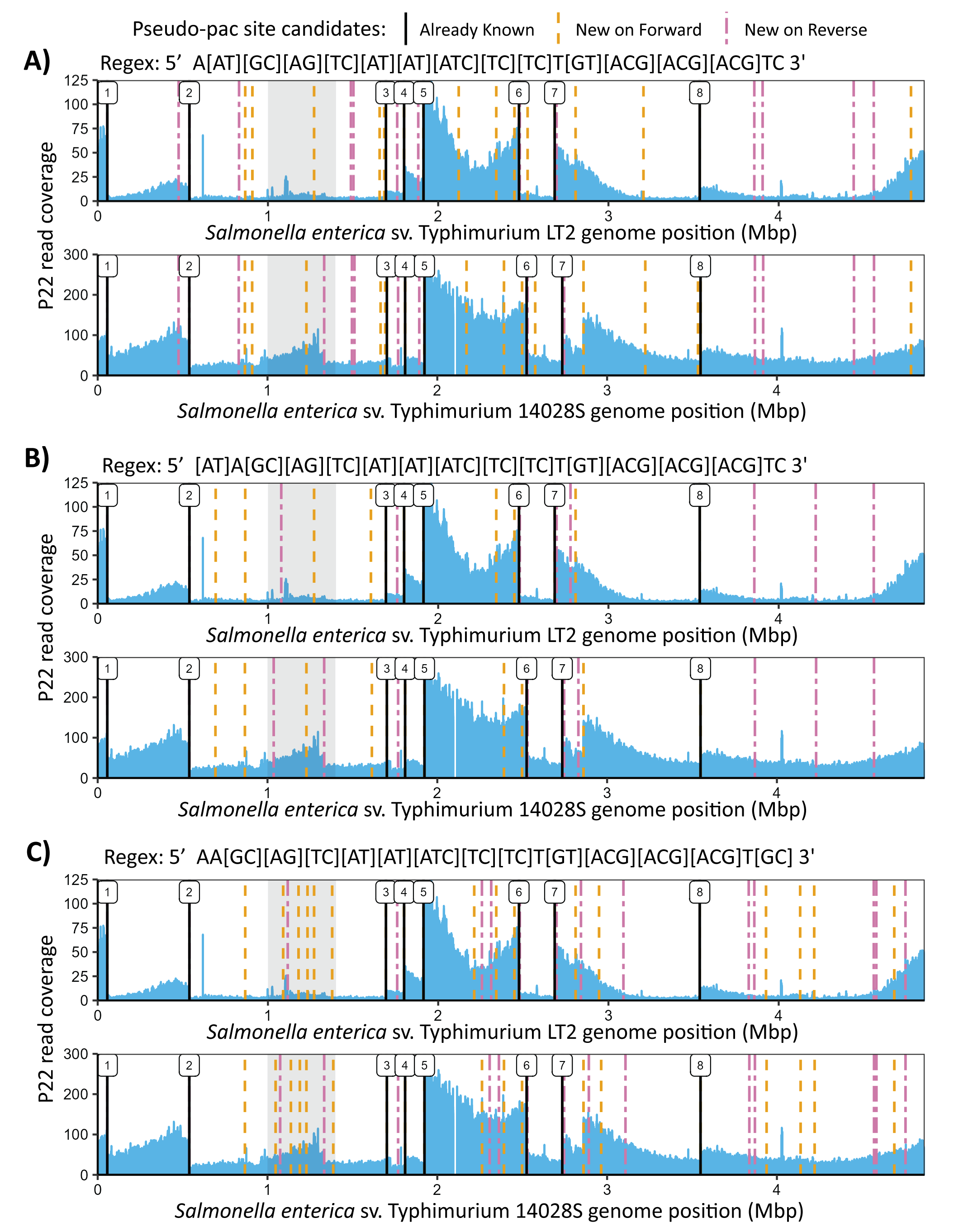

Supplement: S1 Fig — (A–C) Coverage plots of the Salmonella enterica sv. Typhimurium LT2 (LT2) and Salmonella enterica sv. Typhimurium 14028S (14028S) genomes with sequencing reads from purified P22. The additional generalized transduction site present in 14028S but not LT2 is shaded in gray. The regular expression (Regex) patterns used to search the Salmonella genomes for additional pseudo-pac sites are displayed above their associated plots. Black vertical lines indicate the locations of the pseudo-pac site sequences that were previously identified in this study. Orange and pink dashed lines indicate the locations of regular expression matches on the forward and reverse Salmonella genome strands, respectively. (TIF) [file ppat.1012301.s001.tif]
