# Supplementary material for: Pseudo-pac site sequences used by phage P22 in generalized transduction of Salmonella
Source: PLoS Pathog. 2024 Jun 24;20(6):e1012301. doi: 10.1371/journal.ppat.1012301 (PMC11226127; doi:10.1371/journal.ppat.1012301)
Supplement: S1 Text — (DOCX) [file ppat.1012301.s002.docx]

**S1 Text: R code used to identify the pseudo-pac site sequences::**

#Load packages

library(seqinr)

library(dplyr)

library(stringr)

#Import genome files

STLT2_seq <- read.fasta(file = "StyphimuriumLT2_genome.fasta")

ST14028S_seq <- read.fasta(file= "Styphimurium14028S_genome.fasta")

#Generate 120bp subsets of the LT2 genome associated with the increase in read coverage at each generalized transduction initiation site

STLT2_forward_seq <- STLT2_seq$NC_003197.2 %>% as.vector()

STLT2_reverse_seq <- chartr("atgc", "tacg", STLT2_forward_seq)

P22_PacSite_forward_seq <- as.vector(c("a","a","g","a","t","t","t","a","t","c","t","g"))

P22_PacSite_reverse_seq <- rev(P22_PacSite_forward_seq)

site_1_LT2 <- STLT2_reverse_seq[c(56397:56516)]

site_2_LT2 <- STLT2_reverse_seq[c(538714:538833)]

site_3_LT2 <- STLT2_forward_seq[c(1693010:1693129)]

site_4_LT2 <- STLT2_forward_seq[c(1799643:1799762)]

site_5_LT2 <- STLT2_forward_seq[c(1913698:1913817)]

site_6_LT2 <- STLT2_reverse_seq[c(2474628:2474747)]

site_7_LT2 <- STLT2_forward_seq[c(2684274:2684393)]

site_8_LT2 <- STLT2_forward_seq[c(3536251:3536370)]

#Best matches to the 12 bp P22 pac site consensus sequences at each of the eight 120 bp LT2 genome subsets

all_sites_table <- cbind(P22_PacSite_forward_seq)

colnames(all_sites_table)[1] <- "P22PacSite"

for (i in seq(1,8,1)) {

best_match_count <- 0

A <- 1

B <- 12

if (i == 1 || i== 2 || i == 6) {

site <- get(paste0("site_", i, "_LT2"))

repeat{

site_subset <- site[c(A:B)]

match_count <- sum(P22_PacSite_reverse_seq == site_subset)

if(match_count >= best_match_count){

best_match_count <- match_count

best_match <- rev(site_subset)

}

A <- A+1

B <- B+1

if (B == length(site)) break

}

all_sites_table <- cbind.data.frame(all_sites_table, best_match)

colnames(all_sites_table)[i+1] <- paste0("site",i)

}

else {

site <- get(paste0("site_", i, "_LT2"))

repeat{

site_subset <- site[c(A:B)]

match_count <- sum(P22_PacSite_forward_seq == site_subset)

if(match_count >= best_match_count){

best_match_count <- match_count

best_match <- site_subset

}

A <- A+1

B <- B+1

if (B == length(site)) break

}

all_sites_table <- cbind.data.frame(all_sites_table, best_match)

colnames(all_sites_table)[i+1] <- paste0("site",i)

}

}

# Function to search genome sequences for regular expression matches:

regex_function <- function(GenomeStringForward, RegexPattern, RegexPatternReverse){

regex_matches_forward <- c()

regex_matches_reverse <- c()

reverse_seq <- chartr("atgc", "tacg", GenomeStringForward)

X <- 1

Y <- 17

repeat{

site_subset <- GenomeStringForward[c(X:Y)]

site_string <- site_subset %>% toString()

site_string <- gsub(", ", "", site_string)

site_subset_comp <- reverse_seq[c(X:Y)]

site_string_comp <- site_subset_comp %>% toString()

site_string_comp <- gsub(", ", "", site_string_comp)

if (str_detect(site_string, RegexPattern) == TRUE) {

regex_matches_forward <- c(regex_matches_forward, X)

}

if (str_detect(site_string_comp, RegexPatternReverse) == TRUE) {

regex_matches_reverse <- c(regex_matches_reverse, X)

}

X <- X+1

Y <- Y+1

if (Y == length(GenomeStringForward)) break

}

regex_forward_matches <- data.frame(intercepts=regex_matches_forward, names=rep('New on Forward',length(regex_matches_forward)))

regex_reverse_matches <- data.frame(intercepts=regex_matches_reverse, names=rep('New on Reverse', length(regex_matches_reverse)))

return(list(regex_forward_matches, regex_reverse_matches))

}

#search LT2 genome sequence for 17bp regular expression (regex) pattern:

LT2_regex <- regex_function(STLT2_seq$NC_003197.2, "aag[ag][tc][at][at][atc][tc][tc]t[gt][acg][acg][gca]tc", "ct[acg][acg][acg][gt]t[tc][tc][atc][at][at][tc][ag]gaa")

new_on_forward_regexLT2 <- LT2_regex[[1]] #Positions of matches on forward strand

new_on_reverse_regexLT2 <- LT2_regex[[2]] #Positions of matches on reverse strand

#Search 14028S genome sequence for 17bp regular expression (regex) pattern:

ST_14028S_regex <- regex_function(ST14028S_seq$NC_016856.1, "aag[ag][tc][at][at][atc][tc][tc]t[gt][acg][acg][gca]tc", "ct[acg][acg][acg][gt]t[tc][tc][atc][at][at][tc][ag]gaa")

new_on_forward_regex14028S <- ST_14028S_regex[[1]] #Positions of matches on forward strand

new_on_reverse_regex14028S <- ST_14028S_regex[[2]] #Positions of matches on reverse strand
