# Supplementary material for: Pseudo-pac site sequences used by phage P22 in generalized transduction of Salmonella
Source: PLoS Pathog. 2024 Jun 24;20(6):e1012301. doi: 10.1371/journal.ppat.1012301 (PMC11226127; doi:10.1371/journal.ppat.1012301)
Supplement: Attachment — Submitted filename: Response_to_reviewers_FINAL.pdf [file ppat.1012301.s003.pdf]

Response to reviewers

Pseudo-pac site sequences used by phage P22 in generalized transduction of *Salmonella*

Jessie L. Maier , Craig Gin, Ben Callahan, Emma K. Sheriff , Breck A. Duerkop , Manuel Kleiner

We would like to thank the two anonymous reviewers and the Editor for taking the time to consider our manuscript.

Our responses are in italics.

Reviewer #1:
The manuscript by Maier et al. is well written and answers an important question regarding what kind of host DNA sequences does P22 package. Based on analyzing the sequence reads from P22 genome sequencing data the authors were able to detect sequences that can act as pseudo-pac sites. Further, the predicted consensus sequence was refined by analyzing genome sequencing data of P22 on a different host. I have two minor issues that I would the authors to address to improve the presenation, rigor and reproducibility of the data.

We thank the reviewer for their positive feedback!

Major points:

1. None

Minor points:

1. The quality of the figures and the way they are presented. The figures are very pixelated and may be a better representation of the read abundance through out the genome will be make the impact of the paper more evident.

It was not entirely clear to us what figure(s) the reviewer was referring to in this comment. We uploaded the figures as .tif files in 300 dpi resolution as required by the PLOS Pathogen submission guidelines. It is possible that figure resolution was lost in the combined manuscript pdf and this is what the reviewer was referring to. We have double-checked our image files to ensure that even at high zoom level there is no pixilation.

2. Is the illumina sequencing data for 14028S strain published ? If so please include a reference and if not please mention how it was done."

The Illumina sequencing for 14028S is public in the ENA repository. The associated study and sample name are provided in the 'Data Availability' statement. The 14028S genome sequencing is available in the NCBI database and the RefSeq information is provided in the 'Data

Availability' statement included on the submission form. The data availability statement, which will be included in the final article, reads:

- Data availability statement: P22 sequencing reads used for LT2 read mapping were generated in Kleiner et al. (2020) and are available at ENA (Study: PRJEB6941, Sample: SAMEA2690949). P22 sequencing reads used for 14028S read mapping are available at ENA (Study: PRJEB72417, Sample: SAMEA115180785). The reference genomes used for LT2 and 14028S read mapping are from NCBI RefSeq NC_003197.2 and NC_016856.1, respectively.

Reviewer #2:
This study focuses on the detection of pseudo-pac site sequences from Salmonella enterica Serovar Typhimurium. The team used existing sequencing data to identify where the pseudo-pac site facilitated LT2 genome packaging. These discovered sites were then used to identify those of another strain 14028S.

The method used to identify pseudo-pac sites is convincing, and several articles have already described the phenomenon of over-coverage. One way of improving would be to propose a method of defining consensus other than trial and error. However, this would require a change of scale, with the analysis of data from a large number of phages and hosts.

Validation of the in vitro results proposed by the authors using a plasmid is a good way forward and I hope to see these results in a future article.

Major points:

1. No major modification

Minor points:

1. As a non-native English speaker, I can't comment on the English, but it seems perfectly comprehensible to me.

We thank the reviewer for their positive feedback!
